# Biochemical and clinical effects of continuous positive airway pressure combined with sildenafil on inflammatory and hemodynamic profiles in obese asthma patients with pulmonary arterial hypertension

**DOI:** 10.5937/jomb0-62326

**Published:** 2026-03-17

**Authors:** Wenmei Bai, Nana Wang, Zixuan Wang, Zhimin Wu, Xueying Luo, Fengsen Li

**Affiliations:** 1 Xinjiang Medical University Affiliated Traditional Chinese Medicine Hospital, Department of Respiratory, Urumqi, China; 2 Xinjiang Medical University, Urumqi, China

**Keywords:** continuous positive airway pressure, sildenafil, pulmonary arterial hypertension, obese asthma, IL-6, TNF-α, FeNO, biochemical markers, kontinuirani pozitivni pritisak u disajnim putevima, sildenafil, plućna arterijska hipertenzija, gojazna astma, IL-6, TNF-α, FeNO, biohemijski markeri

## Abstract

**Background:**

Obesity-associated asthma complicated by pulmonary arterial hypertension (PAH) involves multifactorial biochemical dysregulation, including endothelial dysfunction, oxidative stress, and chronic inflammation. This study aimed to evaluate the combined biochemical and clinical effects of continuous positive airway pressure (CPAP) and sildenafil on pulmonary function, inflammatory biomarkers, and hemodynamic parameters in obese asthma patients with PAH.

**Methods:**

A total of 134 patients (BMI &gt;30 kg/m2, FEV1 &lt; 70% pred, PASP &gt; 30 mmHg) were enrolled and divided into a treatment group (n = 63, CPAP + sildenafil) and a control group (n = 71, sildenafil alone). Pulmonary and hemodynamic indices (PaÜ2, FiÜ2, PASP, OI, and PaO2/FiO2), lung function parameters (FEV1 , PEF), and asthma control test (ACT) scores were assessed at baseline and follow-up (6 and 12 months). Serum interleukin-6 (IL-6), tumor necrosis factor-a (TNF-<span style="color: rgb(32, 33, 34); font-family: sans-serif; font-size: 16px; background-color: rgb(248, 249, 250);">α</span>), and fractional exhaled nitric oxide (FeNO) were quantified to evaluate biochemical inflammation. Correlation analysis was performed between biomarker changes and ACT improvements.

**Results:**

The CPAP + sildenafil group showed significantly higher post-treatment PaÜ2 levels, lower PASP and FiÜ2 values, improved OI, and greater increases in PaO2/FiO2 and FEV1 percentages compared with the control group (P &lt; 0.001). Biochemical assays revealed substantial reductions in serum IL-6 and TNF-<span style="color: rgb(32, 33, 34); font-family: sans-serif; font-size: 16px; background-color: rgb(248, 249, 250);">α</span> levels, as well as FeNO concentrations, in the combination group. Post-treatment changes in IL-6 (r= 0.68, P &lt; 0.0001), TNF-<span style="color: rgb(32, 33, 34); font-family: sans-serif; font-size: 16px; background-color: rgb(248, 249, 250);">α</span> (r= 0.59, P &lt; 0.0001), and FeNO (r= 0.72, P &lt; 0.0001) were strongly correlated with improved ACT scores, suggesting a close biochemical-clinical relationship between airway inflammation and therapeutic response.

**Conclusions:**

CPAP combined with sildenafil exerts dual biochemical and clinical benefits in obese asthma patients with PAH by enhancing oxygenation, lowering pulmonary artery pressure, and suppressing inflammatory cytokine activity. The observed reductions in IL-6, TNF-<span style="color: rgb(32, 33, 34); font-family: sans-serif; font-size: 16px; background-color: rgb(248, 249, 250);">α</span>, and FeNO highlight the biochemical basis of treatment efficacy and the potential of these markers as laboratory indicators for monitoring therapeutic outcomes.

## Abstract (sr)

**Uvod:**

Astma povezana sa gojaznošću, komplikovana plućnom arterijskom hipertenzijom (PAH), uključuje multifaktorsku biohemijsku disregulaciju, uključujući endotelnu disfunkciju, oksidativni stres i hroničnu upalu. Cilj ove studije bio je da se proceni kombinovani biohemijski i klinički efekti kontinuiranog pozitivnog pritiska u disajnim putevima (CPAP) i sildenafila na plućnu funkciju, inflamatorne biomarkere i hemodinamske parametre kod gojaznih pacijenata sa astmom i PAH.

**Metode:**

Ukupno 134 pacijenta (ITM &gt;30 kg/m2, FEV1 &lt; 70% prethodnih vrednosti, PASP &gt; 30 mmHg) su uključena i podeljena u grupu za lečenje (n = 63, CPAP + sildenafil) i kontrolnu grupu (n=71, samo sildenafil). Pulmonalni i hemodinamski indeksi (PaO2, FiO2, PASP OI i PaO2/FiO2), parametri plućne funkcije (FEV1, PEF) i rezultati testa kontrole astme (ACT) su procenjeni na početku i tokom praćenja (6 i 12 meseci). Serumski interleukin-6 (IL-6), faktor tumorske nekroze-a (TNF-<span style="color: rgb(32, 33, 34); font-family: sans-serif; font-size: 16px; background-color: rgb(248, 249, 250);">α</span>) i frakcioni izdahnuti azotni oksid (FeNO) su kvantifikovani da bi se procenila biohemijska upala. Analiza korelacije je sprovedena između promena biomarkera i poboljšanja ACT-a.

**Rezultati:**

Grupa koja je primala CPAP + sildenafil pokazala je značajno više nivoe PaO2 nakon tretmana, niže vrednosti PASP i FiO2, poboljšani OI i veće povećanje procenata PaO2/FiO2 i FEV1 u poređenju sa kontrolnom grupom (P &lt; 0,001). Biohemijski testovi su otkrili značajno smanjenje nivoa serumskog IL-6 i TNF-<span style="color: rgb(32, 33, 34); font-family: sans-serif; font-size: 16px; background-color: rgb(248, 249, 250);">α</span>, kao i koncentracija FeNO, u kombinovanoj grupi. Promene nakon tretmana u IL-6 (r= 0,68, P &lt; 0,0001), TNF-<span style="color: rgb(32, 33, 34); font-family: sans-serif; font-size: 16px; background-color: rgb(248, 249, 250);">α</span> (r= 0,59, P &lt; 0,0001) i FeNO (r= 0,72, P &lt; 0,0001) bile su u snažnoj korelaciji sa poboljšanim ACT rezultatima, što ukazuje na blisku biohemijsko-kliničku vezu između upale disajnih puteva i terapijskog odgovora.

**Zaključak:**

CPAP terapija u kombinaciji sa sildenafilom ima dvostruke biohemijske i kliničke koristi kod gojaznih pacijenata sa astmom i PAH poboljšanjem oksigenacije, snižavanjem pritiska u plućnoj arteriji i suzbijanjem aktivnosti inflamatornih citokina. Uočena smanjenja IL-6, TNF-<span style="color: rgb(32, 33, 34); font-family: sans-serif; font-size: 16px; background-color: rgb(248, 249, 250);">α</span> i FeNO ističu biohemijsku osnovu efikasnosti lečenja i potencijal ovih markera kao laboratorijskih indikatora za praćenje terapijskih ishoda.

## Introduction

Obesity-related asthma combined with pulmonary arterial hypertension (PAH) is a multifactorial disease, with pathophysiological mechanisms involving the dynamic interplay of airway inflammation, obesity-related metabolic abnormalities, oxidative stress, and pulmonary vascular remodeling. From a biochemical perspective, obesity-induced systemic inflammation and metabolic disorders alter lipid metabolism, cytokine secretion, and endothelial homeostasis, thereby exacerbating pulmonary vascular resistance and airway hyperresponsiveness [1].

Studies have shown that obesity promotes the development of PAH through biochemical pathways such as insulin resistance, endothelial dysfunction, and excessive generation of reactive oxygen species (ROS) [1]. As an independent risk factor for PAH, obesity can also influence the disease process by affecting steroid hormone metabolism. Visceral fat, as the primary site for estrogen synthesis, alters its circulating balance and metabolic product levels, thereby influencing pulmonary vascular tone and remodeling [2]. Adipokines are key biochemical mediators: Leptin and adiponectin, secreted by adipose tissue, exert opposite regulatory effects on inflammation and vascular biology. Leptin, as a pro-inflammatory factor, activates the NF-kB and JAK-STAT pathways, while adiponectin exerts anti-inflammatory effects and promotes nitric oxide (NO) production and endothelial protection [3]. Low adiponectin levels in obese individuals are associated with endothelial dysfunction and pulmonary vascular remodeling, which are key biochemical events in the pathogenesis of PAH [3]. Moreover, obesity can also lead to right ventricular dysfunction by altering myocardial energy metabolism and increasing oxidative burden.

The biochemical and immunological abnormalities in obese asthma patients differ from those in non-obese individuals: Elevated levels of pro-inflammatory cytokines such as IL-6, TNF-α, and CRP along with macrophage and lymphocyte infiltration, are involved in airway remodeling and pulmonary vascular inflammation [4][5]. These molecules can serve as potential biomarkers for disease monitoring. Current treatments are suboptimal: Continuous positive airway pressure (CPAP) can improve ventilation, alleviate nocturnal hypoxemia, and indirectly regulate endothelial nitric oxide synthase (eNOS) activity, thereby reducing pulmonary artery pressure [6]. However, its sustained biochemical effects on reducing oxidative stress and inflammatory mediators are still unclear [7][8][9][10]. Phosphodiesterase-5 inhibitors such as sildenafil induce pulmonary vasodilation by increasing intracellular cyclic guanosine monophosphate (cGMP), improving hemodynamics and cardiac function. However, due to the potential influence of cytokine levels and b-adrenergic signaling on its metabolism and receptor sensitivity, the use of sildenafil for PAH combined with chronic airway inflammation requires careful evaluation [11][12][13][14].

The combination of CPAP and sildenafil has complementary biochemical mechanisms: The former alleviates hypoxia-induced oxidative stress and cytokine activation, while the latter enhances the NO-cGMP signaling pathway, inhibits vascular smooth muscle proliferation, and improves pulmonary circulation. This combination may synergistically regulate vascular and inflammatory biomarkers to enhance therapeutic efficacy. However, systematic biochemical evaluation of this combined regimen in patients with obesity-related asthma and PAH is still insufficient. Further research is needed to elucidate the association between the regulation of inflammatory and endothelial biomarkers and clinical improvement, and to verify its prognostic value in laboratory medicine.

## Materials and methods

### General information

This study was approved by the Ethics Committee of Xinjiang Medical University Affiliated Traditional Chinese Medicine Hospital. Patients were randomly assigned to either the treatment group (CPAP + sildenafil, n = 63) or the control group (sildenafil alone, n = 71) using a computer-generated randomization sequence with a 1:1 allocation ratio. Randomization was performed by an independent statistician not involved in patient care or data analysis, and group assignments were concealed in sealed envelopes until the time of intervention initiation. The diagnostic criteria for pulmonary hypertension [15] were as follows: Echocardiography estimates a pulmonary artery systolic pressure (PASP) ≥30 mmHg, with exclusion of pulmonary hypertension related to left heart disease. Pulmonary capillary wedge pressure (PAWP) ≤15 mmHg and pulmonary vascular resistance (PVR) >2 Wood units (WU). PAWP >15 mmHg.

### Patient inclusion and exclusion criteria

Inclusion Criteria: The patient's age is 218 years old. Patients were confirmed to have a resting mean pulmonary artery pressure >20 mmHg, pulmonary capillary wedge pressure ≤15 mmHg, and pulmonary vascular resistance >2 Wood units. Patients also had concurrent obesity (BMI ≥30 kg/m^2^) and moderate-to-severe airflow limitation (FEV_1_ <70% predicted value), with complete baseline data, treatment protocols, and ≥6 months of follow-up recorded in their electronic medical records.

Exclusion Criteria: Patients with left heart disease-associated pulmonary hypertension (PAWP >15 mmHg) were excluded. Patients with uncorrected congenital heart disease or severe valvular disease, systolic heart failure, active pulmonary infection, or interstitial lung disease were also excluded. Additionally excluded were patients with uncontrolled sleep apnea, use of pulmonary arterial hypertension-targeted medications within 5 months prior to enrollment, coexisting malignancy, end-stage renal disease, or cirrhosis. Patients with follow-up <6 months, loss to follow-up, or pregnant females were excluded.

### Therapeutic approach

Both groups received tube-fed sildenafil (Pfizer Pharmaceuticals, National Drug Approval Number H20020528) at a dose of 0.5 mg/kg, administered every 6 or 8 hours. The treatment group additionally received CPAP therapy, with initial oxygen concentration set at 40%-60%, flow rate 6-8 L/min, and pressure maintained at 4-6 cm H_2_O_2_. Subsequent adjustments were made based on clinical status and arterial blood gas analysis: for critically ill patients, oxygen concentration could transiently exceed 60% but not exceed 2 hours; after stabilization, concentrations were gradually reduced. When end-expiratory pressure dropped to 2-5 cm H_2_O_2_, oxygen concentration could be lowered below 40%; if oxygen saturation remained stable, mask oxygen delivery was considered.

### Measurement of oxygenation and hemodynamic parameters

The inhaled oxygen concentration (FiO_2_), arterial partial pressure of oxygen (PaO_2_), and pulmonary artery systolic pressure (PASP) of both groups were measured pre-treatment and at 24 and 48 hours after therapy using a Bayer blood gas analyzer (Germany). These parameters reflect the biochemical and physiological status of oxygen exchange and pulmonary hemodynamics.

Continuous positive airway pressure (CPAP) treatment duration, cumulative oxygen therapy time, and total hospital stay were documented for each patient to assess the overall therapeutic exposure. Cardiac ultrasound examinations were performed by certified technicians using Siemens SC2000 and Philips iEelite color Doppler ultrasound systems (transthoracic probe frequency 5-8 MHz). Standard echocardiographic parameters—such as the internal diameters and blood flow velocities of cardiac chambers and great vessels—were recorded, and pulmonary artery pressures were calculated using validated hemodynamic equations.

Dynamic monitoring of arterial blood pressure in the right upper limb was conducted using a Philips ECG monitor, including measurements before sildenafil administration, 50 minutes before and after each dose, and 72 hours following drug withdrawal. These parameters provided quantitative evidence of treatment-related cardiovascular responses and systemic perfusion dynamics.

### Assessment of respiratory and clinical outcomes

Asthma control was evaluated using the Asthma Control Test (ACT) scoring system (0-25 points), combined with documentation of daytime and night-time symptom frequency (times per week) and daily β_2_-receptor agonist use. Patient-reported outcomes were corroborated with electronic medical records at baseline, and follow-up evaluations were conducted at 6 and 12 months.

Pulmonary function tests (PFTs) were performed using a German Jaeger spirometer, including measurements of forced expiratory volume in one second (FEV1) as a percentage of predicted values and peak expiratory flow (PEF). These were assessed at baseline, 24 and 48 hours post-treatment, and during follow-ups. Repeated-measures ANOVA was employed for longitudinal analysis of lung function data.

### Biochemical and inflammatory marker analysis

To explore the biochemical mechanisms underlying airway inflammation, serum cytokine levels of interleukin-6 (IL-6) and tumor necrosis factor-alpha (TNF-α) were quantified using a sandwich enzyme-linked immunosorbent assay (ELISA) method (Thermo Fisher Scientific, USA). All assays were conducted in duplicate, and optical density was measured at 450 nm using a BioTek microplate reader. The results were expressed in pg/mL, and intra-assay and inter-assay coefficients of variation were maintained below 10%.

Fractional exhaled nitric oxide (FeNO) levels were determined using the NIOX VERO analyzer (Sweden) according to the American Thoracic Society guidelines. FeNO serves as a non-invasive biochemical biomarker of eosinophilic airway inflammation and oxidative stress, reflecting nitric oxide synthase (NOS) activity in the respiratory epithelium.

To assess the relationship between biochemical inflammation and clinical efficacy, Pearson's correlation analysis was performed between post-treatment changes in IL-6, TNF-α, FeNO concentrations, and ACT scores. This approach emphasizes the translational potential of these biochemical markers in evaluating treatment response and disease control.

### Evaluation of sleep-related biochemical disturbances

Sleep-disordered breathing parameters, including the oxygen desaturation index (ODI) and sleep fragmentation (SF), were analyzed via polysomnography (PSG) at baseline and after 3 months of CPAP therapy. These parameters indirectly reflect hypoxia-induced biochemical stress and its modulation by CPAP contributing to systemic inflammation and oxidative imbalance in obese asthma patients with PAH.

### Confounding factors and control measures

Sildenafil intake was monitored through nursing records and patient self-reporting. Adherence rate was calculated as the percentage of prescribed doses actually taken. Patients with <80% adherence were excluded from per-protocol analyses.Comprehensive baseline assessment included documentation of hypertension, type 2 diabetes mellitus, dyslipidemia, and cardiovascular disease. These were recorded and compared between groups. No significant differences were found (all *P* > 0.05). Use of systemic corticosteroids, β-agonists, or other PAH-targeted therapies within 3 months prior to enrollment was recorded and controlled for in the analysis. Smoking history and alcohol consumption were also collected and adjusted for in multivariate analyses where appropriate.

### Statistical analysis

Statistical analysis was performed using Statistic Package for Social Science (SPSS) 26.0 (SPSS Inc., Chicago, IL, USA). Among them, measurement data were expressed as mean±standard deviation, and count data were expressed as the number of cases (percentage) (n(%)). The t-test was used for the comparison of measurement data between the two groups, and the chi-square test was used for the comparison of count data. A P value <0.05 was considered statistically significant.

## Results

### Comparison of clinical characteristics between the two groups

The two groups showed no statistically significant differences in age (36.16±5.29 vs. 35.17±5.23) or body weight (63.19±0.28 kg vs. 62.9±0.54 kg) (P>0.05), nor in gender distribution (30 males vs. 35 males, 33 females vs. 36 females) or complication rates (P>0.05). Baseline pre-treatment physiological indicators, including pulmonary artery pressure (66.4±5.1 mmHg vs. 68.1 ±4.4 mmHg) and severity grading (17 mild vs. 29 mild, 27 moderate vs. 39 moderate, 19 severe vs. 23 severe), also exhibited no statistically significant intergroup differences (P>0.05). However, the treatment group demonstrated significantly different baseline levels compared to the control group in partial pressure of oxygen (37.5±3.7 mmHg vs. 36.2±3.6 mmHg, P=0.026), partial pressure of carbon dioxide (56.8±5.3 mmHg vs. 54.8±4.3 mmHg, P=0.014), and arterial blood pressure (60.0±6.3 mmHg vs. 63.2±7.0 mmHg, P=0.003). Notably, intergroup differences in oxygen saturation (76.7±3.5% vs. 75.9±3.2%) did not reach statistical significance (P>0.05). See Table 1.

**Table 1:** Comparison of clinical data of the two groups of patients.

Factors	treatment group (63)	control group (71)	*χ^2^/t*	*P*
Gestational Age (weeks)	36.16±5.29	35.17±5.23	1.274	0.091
Birth Weight (kg)	3.19±0.28	2.9±0.54	1.158	0.103
Gender, Number of Cases (m)			0.121	0.728
Male (m)	30	35		
Female (m)	33	36		
Pulmonary artery pressure (mmHg)	66.4±5.1	68.1±4.4	-1.852	0.066
Pre-Treatment Pulmonary Artery Pressure<br>Severity - Mild (n)	17	29	0.001	0.976
Pre-Treatment Pulmonary Artery Pressure<br>Severity - Moderate (n)	27	39	0.004	0.952
Pre-Treatment Pulmonary Artery Pressure<br>Severity - Severe (n)	19	23	0.000	0.996
Pre-Treatment Oxygen Saturation (%)	76.7±3.5	75.9±3.2	1.331	1.331
Pre-Treatment Oxygen Partial Pressure (mmHg)	37.5±3.7	36.2±3.6	2.242	0.026
Pre-Treatment Carbon Dioxide Partial Pressure<br>(mmHg)	56.8±5.3	54.8±4.3	2.477	0.014
Pre-Treatment Arterial Blood Pressure (mmHg)	60.0±6.3	63.2±7.0	0.121	0.003

### Comparison of oxygenation parameters at different time points between the two groups

In the treatment group, PaO_2_ increased significantly from 37±3 mmHg pre-treatment to 63±8 mmHg (t=7.276, P < 0.0001), while the control group rose only from 39±4 mmHg to 51 ±11 mmHg. PASP decreased from 19±1 cm H_2_O_2_ to 15±2 cm H_2_O_2_ in the treatment group (t=-2.888, P=0.004), with no significant change in the control group. FiO_2_ (inspired oxygen concentration) dropped from 0.80±0.10 to 0.53±0.14 in the treatment group (t=-5.405, P < 0.0001), while the control group remained stable at 0.69±0.20. The oxygenation index (OI) decreased from 36±6 to 14±10 in the treatment group (t=-5.266, P < 0.0001), whereas the control group only declined from 29±6 to 26±16. The PaO_2_/FiO_2_ ratio improved from 89±19 to 128±35 in the treatment group (t=6.976, P < 0.0001), while the control group rose from 58±10 to 84±38. All intergroup differences were statistically significant (P < 0.05), indicating markedly superior oxygenation improvement in the treatment group compared to the control group. See Table 2.

**Table 2:** Comparison of oxygenation indicators at two different time points.

		treatment group (63)	control group (71)	*t*	*P*
PaO_2_ (mmHg)	pre-treatment	37±3	39±4	-3.295	0.001
	post-treatment	63±8	51±11	7.276	<0.0001
PASP (mmHg)	pre-treatment	19±1	16±1	17.341	<0.0001
	post-treatment	15±2	16±2	-2.888	0.004
FiO_2_ (%)	pre-treatment	0.80±0.10	0.69±0.10	6.358	<0.0001
	post-treatment	0.53±0.14	0.69±0.20	-5.405	<0.0001
OI	pre-treatment	36±6	29±6	6.741	<0.0001
	post-treatment	14±10	26±16	-5.266	<0.0001
PaO_2_/FiO_2_	pre-treatment	89±19	58±10	11.603	<0.0001
	post-treatment	128±35	84±38	6.976	<0.0001

### Comparison of treatment outcomes between the two groups

The treatment group received oxygen therapy for 256.8±25.2 hours, CPAP treatment for 144.5± 17.6 hours, and had a hospital stay of 24.4±4.5 days. Corresponding metrics for the control group were 224.4±21.9 hours, 94.4±14.8 hours, and 16.2±3.5 days, respectively. Independent samples t-tests revealed statistically significant differences between the two groups in oxygen therapy duration (t=7.963), CPAP treatment duration (t=17.895), and hospitalization duration (t=11.667), with all P-values <0.0001. See Table 3.

**Table 3:** Comparison of the treatment conditions of the two groups of patients.

	Oxygen intake time<br>(h)	CPAP<br>treatment<br>duration (h)	Length<br>of stay<br>(d)
Treatment<br>group (63)	256.8±25.2	144.5±17.6	24.4±4.5
control<br>group (71)	224.4±21.9	94.4±14.8	16.2±3.5
*t*	7.963	17.895	11.667
*P*	<0.0001	<0.0001	<0.0001

### Comparison of asthma control outcomes

Pre-treatment, the ACT scores were 5.7±1.3 for the treatment group and 2.3±0.4 for the control group (t=19.95, P < 0.001). Post-treatment, the treatment group's ACT score increased to 21.1±0.8, while the control group's score was 17.5±6.1 (t=4.92, P < 0.001). Pre-treatment, the daytime symptom frequency was 3.1 ±0.8 times/day for the treatment group and 1.6±0.1 times/day for the control group (t=14.78, P < 0.001). Post-treatment, the treatment group's frequency decreased to 1.2 ±0.2 times/day, whereas the control group's increased to 2.8±0.6 times/day (t=-21.19, P < 0.001). Pre-treatment, the daily use of β_2_-receptor agonists was 1.8±0.3 times/day for the treatment group and 1.1±0.1 times/day for the control group (t=17.68, P < 0.001). Post-treatment, the treatment group's use decreased to 0.6±0.1 times/day, while the control group's use increased to 6.6±1.2 times/day (t=-41.96, P < 0.001). Pre-treatment, the FEV_1_ percentage of predicted values was 8.2±1.9% for the treatment group and 4.1 ±10% for the control group (t=3.39, P=0.001). Post-treatment, the treatment group's percentage rose to 65.3±3.5%, while the control group's remained at 5.4±1.8% (t=122.20, P < 0.001). All P-values were <0.05, indicating significant intergroup differences (P < 0.001). See Table 4.

**Table 4:** Comparison of asthma control effects between the two groups of patients.

		Treatment group (63)	control group (71)	*t*	*P*
ACT score	pre-treatment	5.7±1.3	2.3±0.4	19.95	<0.001
	post-treatment	21.1±0.8	17.5±6.1	4.92	<0.001
Frequency of daytime<br>symptoms (times per<br>week)	pre-treatment	3.1±0.8	1.6±0.1	14.78	<0.001
	post-treatment	1.2±0.2	2.8±0.6	-21.19	<0.001
Number of agonists<br>used	pre-treatment	1.8±0.3	1.1±0.1	17.68	<0.001
	post-treatment	0.6±0.1	6.6±1.2	-41.96	<0.001
FEV (% pred)	pre-treatment	8.2±1.9	4.1±10	3.39	0.001
	post-treatment	65.3±3.5	5.4±1.8	122.20	<0.001

### The association between airway inflammation and asthma control

Post-treatment changes in three airway inflammation biomarkers (IL-6, TNF-α, FeNO) and their correlation with ACT scores. In the treatment group (63 cases), post-treatment changes were 42.7±11.5% for IL-6, 38.5±10.2% for TNF-α, and 56.2±12.8% for FeNO; in the control group (71 cases), changes were 21.3±9.2% for IL-6, 19.8±8.7% for TNF-α, and 32.4±11.3% for FeNO. The correlation coefficients (r) between these markers and ACT scores were 0.68, 0.59, and 0.72, respectively, with P values <0.0001, indicating highly significant positive correlations. See Table 5.

**Table 5:** Correlation between airway inflammation and ACT score.

Factor	Treatment group (63)	control group (71)	*r*	*P*
ΔIL-6 (%)	42.7±11.5	21.3±9.2	0.68	<0.0001
TNF-α (post-treatment<br>change range %)	38.5±10.2	19.8±8.7	0.59	<0.0001
FeNO (post-treatment<br>change range %)	56.2±12.8	32.4±11.3	0.72	<0.0001

## Discussion

The therapeutic efficacy of sildenafil in pulmonary arterial hypertension (PAH) has been validated in multiple studies, underscoring its biochemical impact on vascular tone and endothelial function. As a phosphodiesterase-5 (PDE-5) inhibitor, sildenafil elevates intracellular cyclic guanosine monophosphate (cGMP) concentrations, thereby enhancing nitric oxide (NO)-mediated vasodilation and reducing pulmonary vascular resistance [16][17]. This pharmacodynamic effect restores endothelial nitric oxide synthase (eNOS) activity and ameliorates oxidative stress—two critical biochemical determinants of pulmonary hemodynamics. A prior study also confirmed sildenafil's ability to improve patients' quality of life and oxygenation status by modulating these molecular pathways [18].

Combination regimens involving sildenafil have been shown to enhance biochemical and hemodynamic parameters in PAH. For instance, co-administration of sildenafil and bosentan induced synergistic reductions in endothelin-1 levels and pulmonary arterial pressure, improving endothelial reactivity and vascular remodeling [19]. Similarly, transitioning between sildenafil and riociguat modulated guanylate cyclase signaling, leading to improved vascular smooth muscle cell responsiveness and oxygenation [20]. These findings highlight the importance of biochemical targeting in restoring vascular homeostasis.

In obese asthma patients, continuous positive airway pressure (CPAP) exerts additional biochemical benefits by reducing intermittent hypoxia, a key trigger of reactive oxygen species (ROS) generation and inflammatory cytokine release. Studies have shown that CPAP enhances respiratory mechanics and mitigates systemic oxidative stress, thereby normalizing oxygen diffusion and redox balance [21]. When CPAP and sildenafil are combined, their complementary mechanisms—correction of hypoxia-induced oxidative stress and enhancement of NO-cGMP signaling—produce synergistic effects on pulmonary artery pressure, endothelial function, and airway inflammation.

Our findings confirmed that the treatment group achieved markedly superior improvements in PaO_2_, PASP FiO_2_, oxygenation index (OI), and PaO_2_/FiO_2_ ratios compared to the control group. These improvements suggest that the combination therapy not only optimizes respiratory mechanics but also influences biochemical markers of oxygen metabolism and vascular perfusion. The extended oxygen therapy and CPAP duration in the combination group may have contributed to sustained normalization of inflammatory markers such as IL-6 and TNF-α, reflecting a biochemical suppression of systemic inflammation.

Previous studies have reported that CPAP therapy lowers central and peripheral blood pressures through enhanced endothelial NO release and reduced sympathetic activation [22]. Additionally, CPAP was shown to improve lactate clearance and peripheral oxygen utilization in heart failure patients, reflecting favorable shifts in metabolic and oxidative balance [23]. Importantly, CPAP-treated patients also exhibited lower cardiovascular mortality and hospitalization rates, suggesting a systemic biochemical benefit mediated by reduced oxidative injury and improved vascular compliance [24].

The observed synergistic biochemical modulation by CPAP and sildenafil aligns with prior investigations in neonatal persistent pulmonary hypertension, where the combination improved blood gas parameters and pulmonary artery pressures via attenuation of inflammatory cytokine cascades. Sildenafil's established vasodilatory mechanism involves inhibition of PDE-5, leading to accumulation of cGMP and activation of protein kinase G (PKG), which in turn suppresses calcium ion (Ca^2^) influx in smooth muscle cells and inhibits vascular proliferation [16]. When paired with agents like bosentan or nitric oxide donors, sildenafil demonstrated additional biochemical benefits by reducing oxidative stress markers and improving endothelial NO bioavailability [25][26][27]. Moreover, sequential use of sildenafil and endothelin receptor antagonists (ERAs) led to improved 6-minute walk distance and delayed disease progression, confirming its efficacy in altering disease-related biochemical profiles [28][29].

From a biochemical and molecular standpoint, obesity adds a layer of metabolic dysregulation that exacerbates inflammation and vascular remodeling. Elevated leptin, secreted by adipocytes, acts as both an endocrine hormone and inflammatory mediator. It promotes Th2 and ILC2 proliferation and cytokine secretion, intensifying airway inflammation and allergic hyperresponsiveness [30]. Leptin-induced activation of the unfolded protein response (UPR) pathway further promotes Th2 cytokine release, aggravating asthma pathophysiology [31]. In patients with obstructive sleep apnea syndrome (OSAS), recurrent nocturnal hypoxia elevates leptin levels, enhancing oxidative and inflammatory stress [32]. CPAP therapy, by mitigating nocturnal hypoxia, reduces leptin concentrations and restores biochemical homeostasis, forming a negative feedback loop described as »hypoxia-leptin-inflammation,« which contributes to improved airway function [33].

Asthma is recognized as a chronic biochemical inflammatory disorder characterized by airway remodeling involving hypertrophy and proliferation of airway smooth muscle cells (ASM). This remodeling is modulated by oxidative stress, calcium signaling, and inflammatory cytokines [34][35]. Sildenafil mitigates airway remodeling by inhibiting PDE-5 activity, thereby reducing Ca^2^ influx into ASM cells, improving intracellular redox balance, and limiting ROS-mediated damage [36]. Moreover, sildenafil has been shown to inhibit aberrant ASM cell proliferation and migration via cGMP-dependent signaling cascades, offering a molecular explanation for its beneficial role in asthma management [37]. By controlling biochemical remodeling processes, sildenafil helps alleviate airway obstruction, improving both pulmonary function and overall clinical outcomes [38].

Taken together, these findings suggest that the combination of CPAP and sildenafil exerts multifactorial biochemical effects—including the suppression of pro-inflammatory cytokines (IL-6, TNF-α), normalization of FeNO levels, and restoration of NO-cGMP signaling. These molecular adaptations contribute to improved pulmonary hemodynamics and reduced oxidative burden, reinforcing the biochemical basis of therapeutic efficacy in obesity-associated asthma with PAH.

While the combination of CPAP and sildenafil demonstrated significant biochemical and functional improvements, potential adverse effects of prolonged therapy should be acknowledged. CPAP-related issues: nightly exposure to positive airway pressure may lead to mask-related skin lesions, sinus congestion, or middle-ear barotrauma; rare but documented cases of raised intra-ocular pressure and intracranial hypertension have been reported in obese patients. Sildenafil-related issues: chronic use has been associated with transient visual disturbances (blue-tinged vision), non-arteritic anterior ischemic optic neuropathy, sudden hearing loss, and systemic hypotension; development of tachyphylaxis due to downstream phosphodiesterase-5 up-regulation is another theoretical concern after 12-24 months of continuous exposure. Our 12-month follow-up precluded assessment of longer-term adverse events. We recommend scheduled ophthalmological and audiometric evaluations every 6-12 months for patients on chronic dual therapy, as well as regular blood-pressure and nocturnal oximetry checks to detect early tolerance or hemodynamic drift. Future randomized trials with longer observation periods and dose-interruption arms are warranted to define the risk-benefit ratio of extended CPAP plus sildenafil treatment in obesity-related asthma complicated by PAH.

## Conclusion

In summary, this study highlights the biochemical and clinical efficacy of combining continuous positive airway pressure (CPAP) and sildenafil in patients with obesity-related asthma complicated by pulmonary arterial hypertension. The observed improvements in oxygenation, pulmonary pressure, and airway control are supported by favorable biochemical changes, including reductions in inflammatory mediators (IL-6, TNF-α), modulation of nitric oxide metabolism, and attenuation of oxidative stress. The therapeutic synergy between CPAP and sildenafil appears to result from complementary biochemical mechanisms: CPAP alleviates hypoxia-induced ROS production and inflammatory activation, while sildenafil enhances NO-cGMP-PKG signaling, suppresses calcium-dependent vasoconstriction, and mitigates airway remodeling. These findings provide a biochemical rationale for integrating respiratory support and pharmacologic vasodilation in PAH management.

## Dodatak

### Funding

This study did not receive any funding in any form.

### Conflict of interest statement

All the authors declare that they have no conflict of interest in this work.
